# Spontaneous Vertebral Artery Dissection as the Heralding Manifestation of Previously Undiagnosed Marfan Syndrome in a Young Adult with Posterior Circulation Stroke: A Case Report

**DOI:** 10.3390/reports9030223

**Published:** 2026-07-13

**Authors:** Alawi M. Alkhadrawi, Jawaher Saad, Arwa Alsaleem, Mohammed Al-Hariri, Fayez Alzubair

**Affiliations:** 1 Cardiac Center, King Fahd Hospital of the University, Imam Abdulrahman Bin Faisal University, Al Khobar 31952, Saudi Arabia; alawikd999@gmail.com; 2 College of Medicine, Imam Mohammad Ibn Saud Islamic University, Riyadh 11432, Saudi Arabia; jawher465@gmail.com (J.S.); arwa.s.saleem@gmail.com (A.A.); f.alzubair@ksmc.med.sa (F.A.); 3 Department of Physiology, Collage of Medicine, Imam Abdulrahman Bin Faisal University, Dammam 31441, Saudi Arabia; 4 Heart Health Center, King Saud Medical City, Riyadh 11196, Saudi Arabia

**Keywords:** Marfan syndrome, vertebral artery dissection, cervical artery dissection, cryptogenic ischemic stroke, arterial tortuosity

## Abstract

**Background and Clinical Significance:** Marfan syndrome (MFS) is an autosomal-dominant connective-tissue disorder caused by pathogenic FBN1 variants. Aortic-root dilation and dissection are the canonical complications, whereas spontaneous vertebral artery dissection (VAD) is described only sporadically. Yet, cerebrovascular events are several-fold more common in MFS, and up to 74% of patients exhibit increased vertebral artery tortuosity, a validated predictor of dissection; **Case Presentation:** A 32-year-old African man with hypertension, type 2 diabetes mellitus, tobacco use, and headaches labelled as migraine presented with acute agitation, visual disturbance, vertigo, dysarthria, and right-sided weakness of two hours’ duration. Examination disclosed previously unrecognised marfanoid stigmata: arachnodactyly with positive wrist and thumb signs, reduced upper-to-lower segment ratio, increased arm-span-to-height ratio, dolichocephaly, pectus excavatum, and a high-arched palate. Non-contrast CT showed left occipital and posterior inferior cerebellar hypodensities. CT angiography demonstrated discontinuous intraluminal filling defects in the left vertebral artery at C4 and C2, and MR angiography confirmed long-segment occlusion/stenosis of the intracranial left vertebral artery. Echocardiography revealed mild aortic-root dilation (4.0 cm; Z-score +2.53) and a small patent foramen ovale (PFO) with a positive bubble study. The patient received intravenous thrombolysis followed by antiplatelet therapy, a high-intensity statin, antihypertensive therapy, and intensified glycaemic control. Because the infarct territory matched the dissected vessel and the small PFO carried no high-risk features (RoPE score 6; PASCAL category “unlikely”), VAD was designated the culprit lesion and the PFO incidental; **Conclusions:** Spontaneous VAD may be the inaugural manifestation of unrecognised MFS, antedating aortic complications. In young adults with cryptogenic posterior-circulation stroke and marfanoid features, early cervical imaging and Ghent assessment are warranted, and a coexistent PFO should not be assumed causal. Multidisciplinary evaluation supports accurate attribution and surveillance.

## 1. Introduction and Clinical Significance

Marfan syndrome (MFS) is an autosomal-dominant heritable connective tissue disorder with an estimated prevalence of 1 in 3000–5000 individuals, caused predominantly by pathogenic variants in the FBN1 gene on chromosome 15q21.1, which encodes the extracellular-matrix glycoprotein fibrillin-1 [1]. Loss of fibrillin-1 function impairs microfibrillar assembly and dysregulates transforming-growth-factor-β (TGF-β) signalling, producing a multisystem phenotype that classically involves the skeletal, ocular, cardiovascular, pulmonary, dural, and integumentary systems [2]. The cardinal life-threatening complication is progressive aortic-root dilation culminating in dissection or rupture; consequently, contemporary management algorithms, including the revised Ghent nosology, are weighted heavily toward thoracic-aortic surveillance and prophylactic aortic surgery [2].

Although fibrillin-1 is expressed throughout the arterial tree, extra-aortic vascular complications in MFS have historically received far less attention. A growing body of imaging-based evidence now indicates that abnormal vascular geometry, most prominently increased vertebral and intracranial arterial tortuosity, is highly prevalent in MFS and is mechanistically linked to a heightened risk of spontaneous cervical artery dissection [3,4]. In a landmark cohort, Morris and colleagues demonstrated that approximately 74% of children and young adults with MFS exhibit an elevated vertebral artery tortuosity index, which independently predicted adverse cardiovascular outcomes [3]. Subsequent population-level analyses have shown that hospitalised patients with MFS carry approximately ten-fold increased odds of cervical or cerebral arterial dissection compared with controls [5].

Despite this biological plausibility, spontaneous vertebral artery dissection (VAD) as the first recognised manifestation of MFS remains exceptionally uncommon in the published literature, with only isolated reports over the past three decades [6]. A recent comprehensive review of spontaneous cervical artery dissection concluded that, while the precise contribution of heritable connective-tissue disorders is still debated, MFS is a recurrent and clinically meaningful association, particularly in young adults presenting with cryptogenic stroke [4]. Recognizing this presentation is clinically consequential: it influences acute antithrombotic decision-making, mandates aortic and ocular surveillance, and triggers cascade family screening.

Here we report a young adult man with previously unrecognised MFS in whom a spontaneous left VAD produced an acute multifocal posterior-circulation infarction that coexisted with an incidental fenestrated PFO, and led through systematic physical examination and integrated imaging to a new diagnosis of MFS. We use this case to illustrate (i) the diagnostic reasoning required to discriminate dissection-related stroke from PFO-mediated paradoxical embolism in a young patient; (ii) the mechanistic links between FBN1-driven microfibril pathology, arterial tortuosity, and dissection; and (iii) the practical implications for screening, antithrombotic therapy, and multidisciplinary follow-up. The case is reported in accordance with the CARE guidelines [7].

## 2. Case Presentation

### 2.1. Patient Information and Clinical Timeline

A 32-year-old African man self-presented to the emergency department with the sudden onset, approximately two hours earlier, of blurred vision, vertigo, nausea, slurred speech, and right-sided weakness; he was additionally agitated and intermittently disoriented to time. His past medical history was notable for hypertension of seven years’ duration, type 2 diabetes mellitus of four years’ duration, active tobacco use, and chronic recurrent headaches previously diagnosed as migraine. On targeted questioning about mechanical precipitants, he reported no recent sporting activity, heavy lifting, sexual activity, or sustained or extreme neck positioning; no chiropractic or other cervical manipulation; no recent minor head or neck trauma; and no paroxysms of coughing, sneezing, or vomiting. There was no prodromal neck pain and no headache distinct from his habitual pattern. No family history of MFS, sudden cardiac death, or aortic disease was reported.

### 2.2. Clinical Findings on Presentation

On arrival, the patient was alert, with a Glasgow Coma Scale of 15, but agitated and intermittently irritable. Vital signs were a regular heart rate of 89 beats per minute, blood pressure of 140/85 mmHg, and a respiratory rate of 23 breaths per minute; oxygen saturation was 99% on room air and he was afebrile.

A focused neurological examination was performed. He was oriented to person and place but unsure of the exact date. Speech was mildly dysarthric, with intact naming, repetition, and comprehension. The pupils were equal and reactive, eye movements were full without gaze palsy, and there was a right homonymous hemianopia with macular sparing on confrontation testing (formal automated perimetry, e.g., Humphrey visual-field charting, was not performed in the acute stroke setting); there was no facial droop. Motor testing showed Medical Research Council grade 4/5 power with pronator drift in the right upper and lower limbs, with the face spared, and grade 5/5 power on the left; tone was normal and the right plantar response was extensor. Vibration and proprioceptive sensation were mildly diminished over the right hemibody, with the face spared. On coordination testing there was dysmetria on left finger-to-nose and heel-to-shin testing (on the non-paretic side), ipsilateral to the left cerebellar (posterior inferior cerebellar artery) infarct; gait could not be formally assessed at presentation because of vertigo and truncal instability. Taken together, the deficits localised to the left vertebrobasilar territory and correlated with the responsible lesions as follows: the left occipital (posterior cerebral artery) infarct accounted for the right homonymous hemianopia; the left cerebellar (posterior inferior cerebellar artery) infarct for the ipsilateral (left) limb ataxia and vertigo; and additional left paramedian brainstem (medullary–pontine) ischaemia in the territory of the dissected left vertebral artery for the contralateral (right) pyramidal weakness, the extensor plantar response, and the right medial-lemniscal (proprioceptive and vibratory) sensory loss with facial sparing. This brainstem component lies below the sensitivity of non-contrast CT which is recognised to be insensitive for acute brainstem and posterior-fossa ischaemia and would be expected to be confirmed on diffusion-weighted magnetic resonance imaging (MRI), which was not available (see Section 3.6). The corresponding admission National Institutes of Health Stroke Scale (NIHSS) score was 6 (visual fields, 1; dysarthria, 1; right upper-limb drift, 1; right lower-limb drift, 1; limb ataxia, 1; and right hemisensory loss, 1). No carotid or vertebral bruits were appreciated.

Targeted musculoskeletal and integumentary examination disclosed a previously undocumented marfanoid habitus. The patient was 190 cm tall with an asthenic build; the upper-to-lower segment ratio was reduced and the arm-span-to-height ratio was increased (arm span 200 cm, total height: 190 cm and ratio 1.05).

He exhibited disproportionately long extremities and digits (arachnodactyly) with a positive Walker–Murdoch wrist sign and a positive Steinberg thumb sign. Additional features included a long, narrow skull (dolichocephaly), pectus excavatum, and a high-arched palate noted on intraoral examination.

### 2.3. Systemic Score (Revised Ghent Nosology)

Using the revised Ghent nosology, the patient’s systemic-feature score was itemised as follows: a positive Walker–Murdoch wrist sign together with a positive Steinberg thumb sign (3 points), pectus excavatum (1 point), and a reduced upper-to-lower-segment ratio with an increased arm-span-to-height ratio in the absence of severe scoliosis (1 point) giving a documented systemic score of 5 of 20 points. Dolichocephaly and a high-arched palate were additionally noted but, in isolation, did not reach the three-of-five threshold required to score the craniofacial criterion. On transthoracic echocardiography, the aortic root measured 4.0 cm, corresponding to a Z-score of +2.53 (i.e., ≥2), which fulfils the aortic criterion of the nosology. In a sporadic case without a known FBN1 variant or documented ectopia lentis, the revised Ghent nosology requires aortic involvement together with either a systemic score ≥ 7, ectopia lentis, or a pathogenic FBN1 variant to establish a definitive diagnosis; the present combination of aortic-root enlargement and a systemic score of 5 was therefore regarded as indicating clinically probable MFS. FBN1 sequencing and a formal slit-lamp ophthalmological examination for ectopia lentis were arranged to complete classification but could not be obtained because the patient was subsequently lost to follow-up (see Limitations). Importantly, the diagnosis of vertebral artery dissection rested on vascular imaging and was independent of the recognition of these connective-tissue features; identifying the marfanoid phenotype was nonetheless clinically consequential for prognosis, vascular surveillance, and family screening.

### 2.4. Laboratory Investigations

Key laboratory findings are summarised in Table 1. Haematological indices, renal function, and routine coagulation parameters (INR 1.05; aPTT 28.6 s) were within normal limits, and markers of myocardial injury and systemic inflammation (high-sensitivity troponin I, CRP, ESR) were unremarkable. Metabolic testing indicated poorly controlled diabetes mellitus (HbA1c 9.0%) and an elevated LDL-cholesterol, with mild transaminitis and vitamin D deficiency. A limited thrombophilia and autoimmune screen were negative, comprising a negative antiphospholipid panel with lupus anticoagulant and a negative antinuclear antibody; however, fibrinogen, D-dimer, protein C, protein S, antithrombin III, activated protein C resistance/factor V Leiden, and anticardiolipin and anti-β2-glycoprotein-I antibodies were not assessed, so an inherited or acquired thrombophilia was not formally excluded.

### 2.5. Neuroimaging

Non-contrast cranial CT (Figure 1) revealed acute subacute hypodensities in the left occipital lobe and the territory of the left posterior inferior cerebellar artery (PICA), without intracranial haemorrhage, mass effect, midline shift, or hydrocephalus. Because the posterior inferior cerebellar artery arises directly from the vertebral artery, this infarct pattern localised the responsible lesion to the left vertebral artery rather than to the posterior circulation in general.

CT angiography of the head and neck (Figure 2) showed normal origin and calibre of the bilateral common and internal carotid arteries, the right vertebral artery, and the great vessels at the arch. The left vertebral artery exhibited discontinuous intermittent intraluminal filling defects along its proximal course at the C4 and C2 levels with an abrupt change in calibre, features compatible with arterial dissection complicated by mural thrombus and partial occlusion. The cervical arteries appeared qualitatively tortuous, although a formal vertebral artery tortuosity index was not calculated on the acquired dataset. Magnetic resonance angiography corroborated long-segment occlusion or severe stenosis of the intracranial left vertebral artery. A reduced calibre of the left posterior cerebral artery accompanied, and was concordant with, the left occipital infarct; because the basilar artery and the right vertebral artery were both patent, any apparent reduction in the contralateral posterior cerebral artery was attributed to a constitutional small calibre (variant) vessel rather than to acute global posterior-circulation hypoperfusion.

### 2.6. Cardiac Evaluation

Transthoracic followed by transesophageal echocardiography (Figure 3) was performed to investigate a potential cardiac source of embolism. The left ventricle was non-dilated with low-normal systolic function (estimated ejection fraction 50–55%); no intracavitary thrombus was identified, and the left atrial appendage was free of thrombus on transesophageal interrogation. The aortic root measured 4.0 cm at the sinuses of Valsalva. Indexed to the patient’s body surface area (2.02 m^2^), this corresponded to a Z-score of +2.53, indicating mild aortic-root dilatation without dissection, intimal flap, or significant aortic regurgitation. The right ventricular apex appeared mildly dilated. A small fenestrated PFO was identified, and an agitated-saline contrast study was positive both at rest and during the Valsalva maneuver, confirming a right-to-left interatrial shunt. No atrial septal aneurysm was present and the shunt was small rather than large. Continuous inpatient cardiac telemetry throughout the admission showed sinus rhythm with no atrial fibrillation or other embolic-source arrhythmia; prolonged ambulatory (Holter) monitoring and longer-term loop recording were planned to definitively exclude paroxysmal atrial fibrillation but could not be completed because the patient was lost to follow-up.

### 2.7. Diagnostic Reasoning: Attributing the Stroke to Vertebral Artery Dissection Rather than the PFO

Two potentially stroke-relevant findings were identified: a left VAD and a small PFO. Three converging lines of reasoning supported VAD as the culprit lesion.

Anatomical correlation. The infarct territories involving the left occipital lobe and the left posterior inferior cerebellar region were consistent with posterior circulation ischemia, likely related to the dissected left vertebral artery and its branches.

Formal PFO-causality assessment. The Risk of Paradoxical Embolism (RoPE) score [8] was 6 (age 30–39 years, 4 points; cortical infarct on imaging, 1 point; no prior stroke or transient ischaemic attack, 1 point; with no points for the coexisting hypertension, diabetes mellitus, and current smoking). Because the RoPE instrument was derived in genuinely cryptogenic stroke, its probability estimate is most meaningful when no competing cause is present; here, an anatomically congruent vertebral artery dissection provides such a cause. To incorporate the anatomy and physiology of the shunt, the PFO-Associated Stroke Causal Likelihood (PASCAL) classification was applied: combining a RoPE score in the lower category (≤6) with the absence of high-risk echocardiographic features (the shunt was small rather than large, and there was no atrial septal aneurysm) places the patient in the PASCAL “unlikely” category, that is, the PFO is unlikely to be causally related to the index stroke [9].

Lesion-specific plausibility. Cervical artery dissection is a leading cause of stroke in adults younger than 45 years and, in the presence of a heritable connective-tissue disorder with documented arterial tortuosity, is the most biologically coherent explanation for the event [3,4].

Taken together, the small size of the PFO, the absence of an atrial septal aneurysm, the PASCAL “unlikely” classification, and the anatomical specificity of the infarct argued against paradoxical embolism. The mild aortic-root dilation was deemed a chronic stigma of probable MFS requiring surveillance rather than the source of the acute event.

### 2.8. Interventions and In-Hospital Management

The patient was admitted to a stroke unit for monitored care, antithrombotic therapy, and aetiological workup. Because he presented within the therapeutic window with a disabling deficit and the dissection involved the extracranial vertebral artery, he received intravenous thrombolysis with alteplase, which is considered reasonable for acute ischaemic stroke attributable to extracranial cervical artery dissection [10,11]. Endovascular thrombectomy was not undertaken.

After thrombolysis, antithrombotic therapy was selected. Although the randomised CADISS and TREAT-CAD trials found no overall superiority of either strategy, more recent evidence indicates that anticoagulation may reduce ischaemic events relative to antiplatelet therapy in occlusive dissection with intraluminal thrombus, at the cost of a higher bleeding risk [12]. In this patient the balance favoured antiplatelet therapy in the acute phase: he had just received intravenous thrombolysis (which mandates a 24 h delay and repeat imaging before any anticoagulation), carried an established sizeable posterior-circulation infarct that elevated the risk of haemorrhagic transformation, and had a connective-tissue arteriopathy in which intramural-haematoma extension under anticoagulation is a theoretical concern. He was therefore commenced on antiplatelet therapy rather than anticoagulation; the absence of an identified cardioembolic source on continuous telemetry (sinus rhythm) and of a demonstrable thrombophilia on the available panel was consistent with this approach, although neither paroxysmal atrial fibrillation nor an inherited thrombophilia was formally excluded. We acknowledge that anticoagulation, with an early transition to antiplatelet therapy, would have been a defensible alternative given the occlusive morphology, and that the optimal strategy remains an area of clinical equipoise [11].

Blood pressure was managed according to standard post-thrombolysis parameters, with maintenance below 180/105 mmHg during the first 24 h [11], followed by long-term antihypertensive therapy. An angiotensin-receptor blocker (valsartan 80 mg once daily) was combined with amlodipine 5 mg once daily; an angiotensin-receptor blocker was preferred over an angiotensin-converting-enzyme inhibitor because, in MFS, angiotensin-receptor blockade attenuates the transforming-growth-factor-β signalling implicated in aortic-root pathology and has been shown to limit aortic-root growth [13]. High-intensity statin therapy (atorvastatin 40 mg once daily) was initiated during admission for secondary prevention, in the context of an elevated LDL-cholesterol. Glycaemic control targeted a blood glucose of 7.8–10 mmol/L (140–180 mg/dL), managed initially with insulin and subsequently with metformin.

### 2.9. Outcomes and Follow-Up

At discharge, the patient was ambulatory with no residual neurological deficit (mRS 0–1; NIHSS consistent with 0). He was referred to medical genetics for FBN1 testing, to ophthalmology for slit-lamp examination, and to cardiothoracic surgery for aortic-root surveillance with serial echocardiography and a baseline gated CT or MR aortogram, and family cascade screening was initiated.

## 3. Discussion

This case illustrates three points of clinical and educational importance. First, spontaneous vertebral artery dissection can be the heralding manifestation of previously undiagnosed Marfan syndrome, antedating any aortic event. Second, in young adults with cryptogenic stroke, the simultaneous discovery of a PFO and a cervical arterial dissection should prompt structured causal attribution rather than reflexive endorsement of paradoxical embolism. Third, the case offers a practical opportunity to integrate the molecular biology of FBN1-related arteriopathy with the contemporary stroke-prevention literature.

### 3.1. From FBN1 to Vertebral Artery Tortuosity and Dissection

Fibrillin-1, the protein product of FBN1, is a major structural component of arterial microfibrils that, together with elastin, forms the elastic lamellae of large and medium-sized arteries. Defective microfibril assembly weakens the medial layer, predisposes to vessel-wall fragmentation, and dysregulates latent TGF-β release, with downstream effects on smooth-muscle-cell phenotype and matrix remodelling [2]. The same biology that drives aortic-root aneurysm formation operates throughout the arterial tree and is now understood to underlie the markedly increased vertebral and intracranial arterial tortuosity observed in MFS [3,14]. Tortuosity, in turn, alters wall shear-stress distribution and intramural mechanical loading, creating a permissive substrate on which minor, often unrecognised, biomechanical triggers (sustained head rotation, the Valsalva manoeuvre, or undiagnosed atlanto-axial laxity) can initiate intimal tear and intramural haematoma formation [14].

### 3.2. Cervical Artery Dissection in Marfan Syndrome. A Critical Reading of the Literature

The contemporary literature on cervical artery dissection (CeAD) in MFS conveys an apparent paradox. On the one hand, large administrative-database analyses suggest that hospitalised MFS patients carry an approximately ten-fold increased risk of arterial dissection overall [5]. On the other, the same study did not find a statistically significant elevation in the specific incidence of vertebral artery dissection compared with controls a finding most plausibly explained by under-ascertainment in patients whose cervical vasculature is not routinely imaged [5]. The Morris cohort, which prospectively measured vertebral tortuosity in MFS, provides the strongest mechanistic correlate: an elevated tortuosity index in 74% of patients was independently associated with adverse cardiovascular events [3]. A 2024 report by Vornetti and colleagues described atlanto-axial dislocation as a precipitant of VAD in MFS, highlighting craniovertebral-junction laxity as an additional dissection substrate that warrants targeted imaging [15]. The 2023 review by Gunduz and colleagues, the most comprehensive contemporary synthesis, concluded that although heritable connective-tissue disorders remain a minority cause of spontaneous CeAD, MFS is a recurrent, biologically coherent, and clinically relevant association, particularly in younger patients [4].

### 3.3. Therapeutic Implications: Antithrombotic Strategy in Cervical Artery Dissection

Optimal antithrombotic therapy for symptomatic cervical artery dissection remains an area of active investigation. The two pivotal randomised trials, CADISS [16] and TREAT-CAD [17], did not demonstrate the superiority or formal non-inferiority of antiplatelet therapy versus vitamin-K-antagonist anticoagulation for prevention of recurrent stroke or major bleeding in the early phase. The 2024 American Heart Association scientific statement on the treatment and outcomes of CeAD in adults [18] recognizes both as acceptable first-line strategies, individualised according to dissection morphology (occlusive versus non-occlusive), the presence of intraluminal thrombus, infarct burden, and bleeding risk. More recent data sharpen this picture: in the STOP-CAD study, anticoagulation was associated with a significantly lower risk of subsequent ischaemic stroke, specifically in patients with occlusive dissection (though with a higher risk of major haemorrhage when continued to 180 days) [12], and a systematic review and meta-analysis similarly found lower ischaemic-stroke rates with anticoagulation, offset by increased bleeding [19]. These data informed the individualised decision in the present case and explain why anticoagulation would have been a reasonable alternative. In MFS, where vessel-wall fragility may theoretically heighten the risk of intramural-haematoma extension, this risk–benefit balance warrants explicit discussion with the patient. Antithrombotic therapy is typically maintained for three to six months, followed by reassessment of vascular healing with non-invasive imaging [18].

### 3.4. The PFO Coexistence: A Lesson in Diagnostic Discipline

PFO is present in approximately one in four healthy adults; its discovery in a young stroke patient therefore does not automatically establish causation. The RoPE score provides a probability-weighted estimate of whether a PFO is causal or incidental in cryptogenic stroke [8], and the complementary PASCAL classification refines this by integrating high-risk shunt features (a large shunt or an atrial septal aneurysm); in the present case a RoPE of 6 with no high-risk features yielded a PASCAL classification of “unlikely” [9]. Importantly, both instruments assume the absence of an alternative competing mechanism and the demonstration of an anatomically congruent dissection effectively closes the causal loop in this case. Closure of the PFO is therefore not indicated; surveillance and management should focus on the dissected vessel and the underlying connective-tissue disorder.

### 3.5. Clinical Recommendations

Five practical lessons emerge. First, every young patient with cryptogenic stroke deserves a careful examination for stigmata of heritable connective-tissue disease, including arm-span measurement, wrist and thumb signs, palatal inspection, and ophthalmological referral. Second, when MFS is suspected or confirmed, cervical and intracranial vascular imaging should be added to the standard aortic-surveillance protocol, with attention to the vertebral artery tortuosity index. Third, antithrombotic therapy must be individualised; current evidence does not mandate anticoagulation over antiplatelet therapy, but explicit shared decision-making is essential, particularly when vessel-wall fragility is suspected. Fourth, aggressive control of modifiable vascular risk factors is essential in this patient, blood-pressure optimization, intensive glycaemic control (admission HbA1c 9.0%), high-intensity statin therapy for an elevated LDL-cholesterol, and structured smoking-cessation, both to support lower recurrent-stroke risk and because these factors compound the arteriopathy of MFS. Fifth, the diagnosis triggers cascade family screening, ophthalmological evaluation, and lifelong aortic surveillance, a workflow that requires coordinated care across neurology, cardiology, medical genetics, ophthalmology, and primary care.

### 3.6. Limitations

Several limitations should be acknowledged. This is a single-patient observation and therefore cannot establish causality between MFS and VAD beyond biological plausibility and consistency with the broader literature. Diagnostic classification of the connective-tissue disorder was incomplete: *FBN1* sequencing and a formal slit-lamp examination for ectopia lentis could not be obtained because the patient was lost to follow-up, so the diagnosis rests on the combination of aortic-root dilatation and a systemic score of 5 points, and is best regarded as clinically probable rather than genetically confirmed Marfan syndrome. The thrombophilia work-up was also incomplete (protein C, protein S, antithrombin III, activated protein C resistance/factor V Leiden, and anticardiolipin/anti-β2-glycoprotein-I antibodies were not measured), and prolonged cardiac monitoring to exclude paroxysmal atrial fibrillation was not completed; these gaps temper the attribution of a non-cardioembolic, non-thrombophilic mechanism. The optimal antithrombotic strategy is debated, and anticoagulation would have been a reasonable alternative to the antiplatelet therapy chosen. From an imaging standpoint, diffusion-weighted imaging and fat-suppressed axial T1-weighted cervical MRI, which are considered reference standard sequences for demonstrating intramural hematoma, would have further strengthened the diagnosis of arterial dissection had they been available. Diffusion-weighted MRI would, likewise, have been expected to demonstrate the small left paramedian brainstem infarct that was inferred clinically from the contralateral (right) pyramidal and medial-lemniscal signs, but was not resolved on the non-contrast CT; this radiologically occult brainstem component and the absence of formal visual-field perimetry are acknowledged limitations of the acute work-up. Posterior-circulation infarct burden was not formally graded; it can be quantified with the posterior-circulation Alberta Stroke Program Early CT Score (pc-ASPECTS), which was not calculated prospectively in this acute single-patient setting and is recommended for future cases. Finally, vertebral-artery tortuosity-index measurement was not performed; given its mechanistic relevance, retrospective measurement on the existing CT-angiography dataset is encouraged.

### 3.7. Future Directions

Prospective registries that systematically screen MFS cohorts for cervical and intracranial arterial tortuosity, dissection, and aneurysm formation are needed to define the true incidence of cerebrovascular events in this population. Genotype–phenotype correlations linking specific FBN1 variants with arterial tortuosity and dissection susceptibility would refine individualised surveillance. Finally, dedicated trials of antithrombotic therapy in CeAD patients with heritable connective-tissue disorders, a subgroup systematically excluded from CADISS and TREAT-CAD, would address the most pressing knowledge gap exposed by this case.

## 4. Conclusions

Spontaneous vertebral artery dissection can be the inaugural clinical manifestation of previously unrecognised clinically probable MFS and should be actively considered in any young adult presenting with posterior-circulation ischaemic stroke particularly when tall stature, arachnodactyly, or arm-span-to-height disproportion is evident. The dissection itself is diagnosed on vascular imaging, independent of the connective-tissue phenotype; recognizing the phenotype is what redirects care toward aortic and ocular surveillance, individualised antithrombotic therapy, and family screening. In this setting, a coexistent patent foramen ovale should not be assumed to be causal without anatomical and probabilistic corroboration; the RoPE score and PASCAL classification provide a structured framework, but anatomical correlation between the infarct territory and the dissected vessel is the definitive arbiter. Integrated neurology, cardiology, ophthalmology, and medical-genetics evaluation supports correct attribution, individualised antithrombotic therapy, longitudinal aortic surveillance, and cascade family screening, and offers the best opportunity to convert an acute cerebrovascular event into a lifetime of prevention.

## Figures and Tables

**Figure 1:**
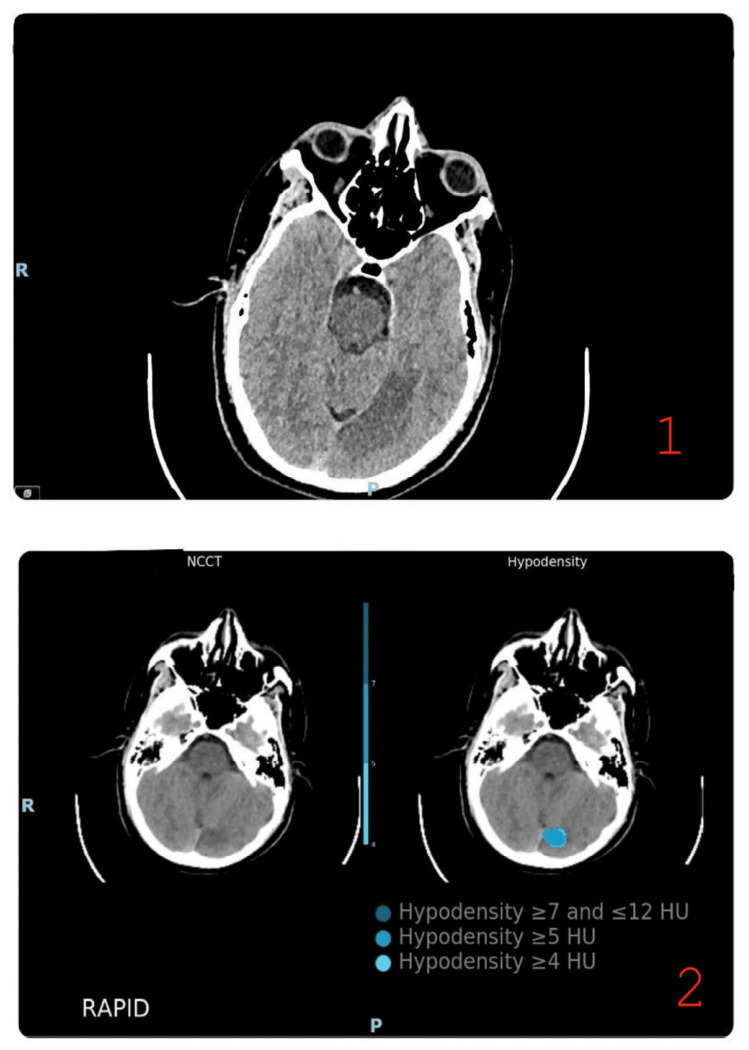
Non-contrast head CT of acute infarction in the left vertebral artery territory. Axial non-contrast CT images demonstrate hypodense areas in the left occipital lobe (**1**) and left posterior inferior cerebellar artery territory (panel (**2**)), highlighted by automated RAPID hypodensity mapping, consistent with acute/subacute infarction. No intracranial hemorrhage or mass effect is present.

**Figure 2:**
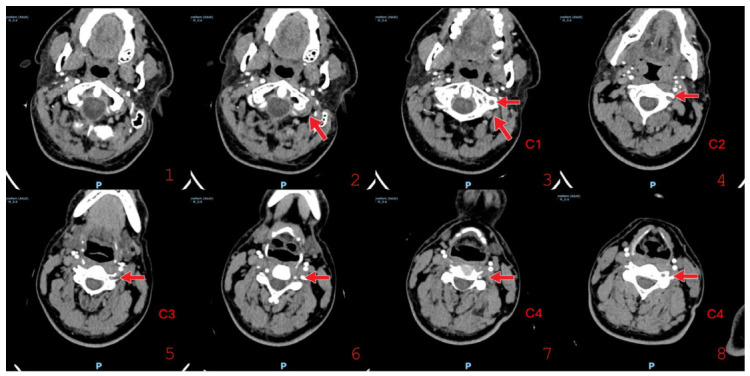
CT angiography of the head and neck demonstrating left vertebral artery dissection. Serial axial CT-angiographic source images are shown, ordered craniocaudally across the C1–C4 vertebral levels (panels (**1**–**8**)). In panel 1 the right vertebral and both carotid arteries opacify normally. In panels (**2**–**8**) the red arrows indicate the abnormal left vertebral artery at successive levels (labelled C1–C4), marking discontinuous intraluminal filling defects and an abrupt change in luminal calibre, consistent with a flow-limiting dissection complicated by mural thrombus.

**Figure 3:**
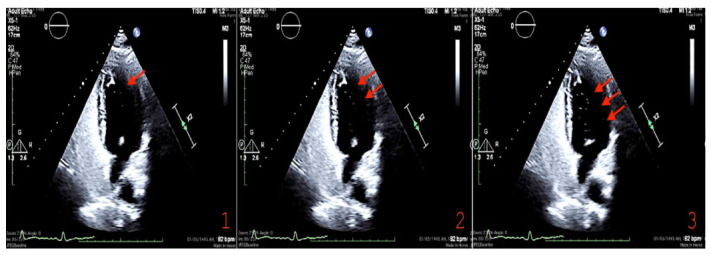
Transthoracic echocardiography with positive bubble study. Apical four-chamber views obtained after agitated-saline injection are shown. Panel (**1**) is an early frame before left-heart opacification; in panels (**2**,**3**) the red arrows indicate microbubbles appearing within the left heart within three cardiac cycles, confirming right-to-left interatrial shunting. The patent foramen ovale itself was directly visualised on the subsequent transoesophageal study.

**Table 1:** Summary of laboratory findings.

Test	Result	Reference Range
Hb (Haemoglobin)	14.4 g/dL	(13–17)
WBC	7.79 × 10^9^/L	(4–10)
Platelets	313 × 10^9^/L	(150–400)
Creatinine	64 µmol/L	(62–106)
Glucose	7.28 mmol/L (H)	(4.11–5.89)
HbA1c	9.0% (H)	(4.8–5.9)
LDL-C	3.73 mmol/L	(Optimal: <2.59)
INR	1.05	(0.8–1.2)
aPTT	28.6 s	(26–40)
Troponin I hs	0.005 µg/L	(<0.014)
CRP	4.73 mg/L	(<5)
Antiphospholipid panel	Lupus anticoagulant	Negative
ANA	Negative	
Vitamin D	43.8 nmol/L (deficiency)	(≥75 normal)
Vitamin B12	962.7 pmol/L (H)	(145–569)
ESR	16 mm/h	(0–30)
ALT	115.7 U/L (H) initially	(0–41)
AST	66.4 U/L (H) initially	(0–40)

Hb: haemoglobin; WBC: white blood cell count; LDL-C: low-density lipoprotein cholesterol; INR: international normalised ratio; aPTT: activated partial thromboplastin time; hs-troponin I: high-sensitivity troponin I; CRP: C-reactive protein; ANA: antinuclear antibody; ESR: erythrocyte sedimentation rate; ALT: alanine aminotransferase; AST: aspartate aminotransferase; HbA1c: glycated haemoglobin.

## Data Availability

All relevant clinical and imaging data supporting the findings of this report are presented within the article. Additional de-identified data are available from the corresponding author on reasonable request.

## Abbreviations

ALT	Alanine aminotransferase
ANA	Antinuclear antibody
aPTT	Activated partial thromboplastin time
AST	Aspartate aminotransferase
CADISS	Cervical Artery Dissection in Stroke Study
CARE	CAse REport (guidelines)
CeAD	Cervical artery dissection
CRP	C-reactive protein
ESR	Erythrocyte sedimentation rate
FBN1	Fibrillin-1 gene
Hb	Haemoglobin
HbA1c	Glycated haemoglobin
INR	International normalised ratio
LDL-C	Low-density lipoprotein cholesterol
MFS	Marfan syndrome
MRI	Magnetic resonance imaging
mRS	Modified Rankin Scale
NIHSS	National Institutes of Health Stroke Scale
PASCAL	PFO-Associated Stroke Causal Likelihood
PCA	Posterior cerebral artery
pc-ASPECTS	Posterior-circulation Alberta Stroke Program Early CT Score
PFO	Patent foramen ovale
PICA	Posterior inferior cerebellar artery
RoPE	Risk of Paradoxical Embolism
STOP-CAD	Antithrombotic Treatment for Stroke Prevention in Cervical Artery Dissection (study)
TGF-β	Transforming growth factor-β
TIA	Transient ischaemic attack
TREAT-CAD	Aspirin versus Anticoagulation in Cervical Artery Dissection (trial)
VAD	Vertebral artery dissection
WBC	White blood cell (count)
